# Annotations

**Published:** 1892-04-23

**Authors:** 


### Annotations.

**Lobworms and their Work.** Mr. Darwin, a few years before his death, made the non-scientific world familiar with the work of worms in passing earth through their bodies, and with the wonderful results effected by them in a comparatively short space of time. More recently Mr. C. Davison has followed up Mr. Darwin's researches in this field of science. Last year Mr. Davison examined the sands between Holy Island and the coast of Northumberland, a large flat stretch of beach familiar to most persons who travel by the East Coast route to Scotland. The observer found that the number of castings of sand thrown up by the lobworms gave an average of 50,000,000 to the square mile. A portion of the castings was weighed, and the total weight thrown up annually was thus shown to be, in some places, about 901 tons per acre; at other points it was a good deal less than this; but in still other parts it amounted to no less than 3,146 tons per acre. If all the sand thus passed through the bodies of these animals in the course of twelve months were spread out it would give an average thickness of not less than thirteen inches. How many and various are the changes produced in the sand by the wonderful activity of these industrious worms it is impossible to say. But it is easy to see how the presence of such creatures in large numbers operates with other forces to produce a kind of order on the surface of the earth, and to replace crudeness by beauty. The thing that strikes one most, however, is the magnitude of the results which can be produced in a short time when a large number of separate individuals work in co-operation by the same methods towards the same end.

**The New Time Coming.** Great is scientific truth, and must prevail. Mr. Clarence King, of the United States Geological Survey, contributes an article to the *Forum* on the Education of the Future. Mr. King sees, what doctors have long seen, that the mass of the population must either take account of the facts and teachings of physiology, or civilization will go backwards instead of forwards in every department of practical activity. The keener the struggle for life, the more is taken out of the survivor: and the only way by which the strife can be successfully maintained is by better breeding, better training, and a more intelligent utilisation and conservation of health and energy. Says Mr. King, "If the future of man's mechanical industry lies under the shadow of the laws of energy, the future of his whole bodily nature, its health, beauty, and organic purity, its strength of muscle, nerve, and brain, depend upon intelligent obedience to the new table of biological commandments. In his ignorance of human biology, man has done little or nothing to protect society from the fatal percentage of disease, crime, and incompetence. Like a patient beast of burden, humanity has staggered since Eden under a load of ills, nearly all of which might have been prevented by a vigorous application of scientific biological restraints. We have been quick to adopt railways, but we cannot realise heredity; we have eagerly put our ear to the telephone, and been wilfully deaf to the voice of science, which is offering to tell us how to make our own children strong and fair." This somewhat rhetorically expressed dictum is not only true, but rather understates than overstatesthe truth. There are few except specialists who have any real appreciation of the possibilities that lie open to a right use of physiological knowledge. Unhappily, the specialist is too often a miser; he buries his knowledge in the secret recesses of his own brain, instead of scattering the seed all over the world to fructify a hundredfold. This he does because he has accepted narrow, illiberal, and foolish traditions from his half-educated scientific ancestors. But the pedantic phase of science will pass, is now fast passing indeed, and the light of physiological truth will yet shine into every corner of the world. It is of excellent omen that men like Mr. Clarence King see so clearly, and point out so precisely and resolutely the way in which the present and future generations must walk if they are to save both their bodies and their souls alive.

**Lady Paget on Vegetarianism.** Lady Paget seems to be determined to constitute herself an authority on matters medical. Having done her best to fill the world with the fame of Count Mattei, she is now bent upon rendering a similar service to vegetarianism. In this, however, she is obliged to venture into the region of theory, and if her Matteist "facts" have no sounder basis than her vegetarian theories possess, medical men will understand that their value is decidedly microscopical. "It is certain," says Lady Paget, "that the giving up of animal food cures many illnesses which no medicine can reach." The obvious reply to this is, that it is certain that medical men would like to know exactly what those diseases are, and also what kind of evidence Lady Paget has for her certainty. "Everybody knows," says Lady Paget, "the bad effect of butchers' meat in gout and rheumatism." But what everybody knows is by no means always the whole truth, or even the truth at all. Too much butchers' meat is bad for gouty people, and indeed, for all other people. But many persons who suffer from gout, would suffer much more if they took no meat at all; and a large proportion of rheumatic people absolutely require an exceptionally nutritious meat diet. "If one reflects," says Lady Paget, "that whilst the meat-eaters' heart has seventy-two beats in the minute the vegetarian's has only fifty-eight beats, therefore 20,000 beats less in the course of the twenty-four hours. Insomnia and nervousness are affected in the same way; there is less wear and more repose in the constitution." Never was there a more fallacious piece of reasoning. As a matter of fact, persons whose pulses beat seventy-two to the minute are, as a rule, infinitely more strong, healthy, bright, and intellectually and physically capable than those whose pulses beat only fifty-eight to the minute. To push Lady Paget's argument to its logical issue, it is only necessary to consider that if fifty-eight pulse beats are better than seventy-two, forty would be better than fifty-eight, twenty better than forty, and none at all better than twenty, and that would be death; *quid est absurdum.* Does Lady Paget seriously believe that a stagnant pool is better than a running stream; that a horse whose paces are only equal to eight miles an hour has a finer constitution than a champion that can trot twelve? But medical men need not quarrel with Lady Paget. She offers them some delightfully foolish hypotheses, the knocking down of which helps the establishment of the truth.

